# Global Access to Health Care and Well-Being: A Place for Policy and Science

**DOI:** 10.3389/fpubh.2016.00129

**Published:** 2016-06-28

**Authors:** Ladislav Záliš, Áine Maguire, Kristen Soforic, Kai Ruggeri

**Affiliations:** ^1^Department of Psychology, Faculty of Social Studies, Masaryk University, Brno, Czech Republic; ^2^Policy Research Group, Department of Psychology, University of Cambridge, Cambridge, UK; ^3^Health Care Department, NORC at the University of Chicago, Chicago, IL, USA; ^4^Engineering Design Centre, Department of Engineering, University of Cambridge, Cambridge, United Kingdom

**Keywords:** medical travel, global health, health-care access, well-being, health policy, measurement, health services, health economics

In contrast with medical tourism, which broadly describes the movement of individuals for non-urgent and unnecessary treatments or recreational and leisure purposes such as cosmetic surgeries or spa visits (1), medical travel explicitly refers to patients crossing national borders with the purpose of receiving treatment that has been determined as essential to maintaining quality of life by a health professional, but may not need to be performed urgently (2). While medical travel offers potential economic gains as well as increased access to health care, it also poses notable risks to the well-being of the patient, which if disregarded could undermine the benefits that patients seek by traveling for medical care. We provide evidence that psychosocial factors such as stress, communication, and social support are integral to understanding the experiences of medical travelers and should be included in the evaluation of treatment outcomes and the development of policies regulating medical travel. We outline several key risks to well-being posed by medical travel, offer preliminary considerations for measuring well-being in outcome studies, and conclude with recommendations for incorporating well-being provisions into medical travel policies.

## Context

With a significant number of people traveling abroad for medical care each year, there is a potential opportunity to control medical spending and increase access to health care by introducing patients to new locations with better quality care, lower costs, or reduced waiting times (2–4). Research has recently begun to establish relevant evidence and put forward considerations, which would support the development of medical travel policies that could help utilize this opportunity (5–7). However, this apparent increase in evidence is predominantly focused on medical or economic gains, and often fails to consider the potential impacts that medical travel might hold for health, beyond treating illness.

It is increasingly recognized by the international community that health and well-being are critical to developing, implementing, and evaluating policies (8–10). This approach, commonly referred to as Health in All Policies, recognizes that health extends beyond merely the absence of illness but rather refers to a state of physical, mental, and social well-being and thus lies outside of simple treatment (8). Accordingly, in response to the increasing numbers of medical travelers, a global health access policy, which considers broader health implications and well-being, particularly in the evaluation of outcomes, may be required.

It has been well demonstrated that a lack of access to appropriate medical care may negatively affect health and well-being (11, 12). For example, a large study in a Chinese population showed that access to health care significantly increases the odds of healthy survival characterized by both high physical and mental well-being (13). The negative impact of poor access to health care on well-being has been observed as particularly strong in developing countries but is also an issue for developed regions (14, 15). While high costs and poor quality are often barriers to health care in developing countries, among developed countries, sources of poor access tend to vary extensively (14, 15). There are major differences in perceived ability to afford care and receive the most effective care as well as disparities in waiting times (16). For instance, not being able to see a doctor due to inability to pay negatively affects well-being in the US, even for people with high income (17). Through improved utilization of available resources across the globe, medical travel can establish different pathways to increasing access to essential treatment.

However, medical travel creates myriad issues beyond those in traditional health-care settings that may impact well-being. These need to be considered before patients are advised to travel abroad for care and included in wider evaluations of such policies. Given that patients traveling abroad for medical care might be in a vulnerable position – ill, traveling to a potentially unfamiliar country or culture, possibly without social support – it is essential to identify the potential risks to medical travelers as well as ways to proactively ensure and promote their well-being at all stages of care. Many of these risks are potentially relevant to all medical travelers, even though some might be particularly pertinent to those patients that are traveling outside of their cultural and geopolitical regions or origins.

## Threats to Well-Being

Patients with illnesses or other disabling conditions are in an inherently stressful position. Such stress may be compounded if they are unfamiliar with hospital settings and events related to their treatment (18). However, when receiving treatment in their own country, policies and best practice guidelines that minimize distress are typically in place. For non-emergency interventions, the care pathway is typically structured, and patients are relatively prepared for the procedure. In most cases, they would have met their treatment provider, attended appointments, and be assured that their medical records are available to the treatment providers. The opportunity to have close relatives or friends with them in the treatment setting, as well as during rehabilitation and recovery, is likely available, and is an instrumental driver of successful recovery (19–23).

Given that medical travel is defined by the movement of patients across international borders, it is not surprising to find critical differences in the experience of acquiring medical treatment at home or abroad. Such differences that existing policies in the treatment country cannot be expected to account for. However, medical travelers are often inadequately informed of the differences associated with receiving care abroad and possibly even presented with biased and incomplete information (24–26). Continuity of information has also been previously reported as a serious issue within cross-border care (26, 27). It is difficult for patients to anticipate their experience when traveling abroad for care: the clinicians, languages, locations, and potentially cultures can be unknown and unfamiliar (28). Sociocultural and language barriers between health-care professionals and patients can impair communication between parties, which predicts decreased patient satisfaction and negative treatment outcomes (29–31). For example, a study of Latino patients in the US showed that language barriers might be a driver of patient dissatisfaction, lower quality of care, and poor health outcomes (32). Furthermore, the availability of social support is substantially diminished for medical travelers, while increased burden is placed on informal caregivers. During medical travel, the informal caregiver is required to fill various important roles encompassing facilitation of information flow between patients and health-care providers, provision of emotional and physical care, coordination of often complex medical travel, and contact between the patient and their broader social network in their home country (24).

These differences are of substantial importance when considering how well-being may be affected by engaging in medical travel. However, at present, approaches to the measurement of well-being in the context of medical travel are limited and inconsistent. In order for policy makers to make informed decisions about the inclusion of well-being in the development of a global health access policy, greater evidence needs to be established. To this end, psychology and other sciences can play an important role in developing adequate approaches to measuring well-being in the context of medical travel and establishing an evidence base to support its inclusion in a health access policy.

## Measurement

Within the context of medical travel, well-being should be measured in terms of its various facets (physical, subjective, and social) using psychometrically valid instruments that are sensitive to change (33). This measurement ought to take place within a framework that focuses on individuals and their support networks and could potentially expand to include the communities that are participating in medical travel. Through this, the impact of medical travel on well-being can be assessed, and further development and calibration of a health care access policy can be considered.

Evaluations of medical traveler outcomes are limited. To date, they focus mainly on patient satisfaction and clinical outcomes for select procedures or localities (7). Medical staff (34–36), facilities (36), support services (34, 35), and continuity of care (37) have been identified as some of the main determinants of patient satisfaction. Although limited in scope and design, evidence from clinical outcome studies suggests that medical tourists may be at increased risk for infection and microbial resistance (38–42), complications (43, 44), and mortality (45) compared with those who receive treatment locally. However, the available research on clinical outcomes lacks the systematic, randomized design that characterizes standard outcome studies and fails to provide follow-up data on patients after they return to their home countries (7). Moreover, although some research on patient satisfaction includes measures related to well-being such as the availability of support services, no attempts have been made to explicitly measure the psychosocial dimensions of medical travel alone or in relation to other outcomes.

Assessing individual well-being before and during medical travel may allow for a more individualized approach toward patient needs and help direct resources in response to these needs. Of broader consequence, assessing change in patient well-being before and after medical travel could help to evaluate the direct effects of the entire intervention. As such, there is considerable need for robust evidence using controlled trials to assess the long-term impact on health and well-being for those who received treatment abroad compared with those who received the same treatment in their own country, and those on long-term waitlists. It should also be emphasized that different instruments and methods may be required to assess short- and long-term changes (33).

Combining reliable and validated measures with more qualitative data gathering, such as feedback questionnaires and patient and carer interviews, will add further depth to policy discussions. It will allow policy makers and researchers to identify key issues related to well-being; aspects of the medical travel process that have greatest impact, either positive or negative, on the patient and their carers; and identify whether well-being is in fact a concern for a global policy on medical travel. This will facilitate the calibration of a more effective health access policy that is based on individual consequences and potentially societal impact.

## Recommendations

Myriad arguments for and against medical travel are available, and the numbers of patients traveling abroad for medical treatment appears to be increasing regardless (2). Currently, medical travel is subject to minimal regulation or standardization, and many of its limitations and risks go beyond those addressed in treatment countries’ existing policies and guidelines (6). This inevitably calls for a coherent global health access policy that ensures the adequate provision of care, and considers the impact of medical travel on the health and well-being of patients and other stakeholders.

Although there are certainly economic, legal, and quality considerations to be made when developing policies for medical travel in order to ensure as many benefits and little risks as possible, well-being should not be overlooked. Policy makers should consider the impact of a global health access on the well-being of individuals and communities.

In light of the principled debates related to medical travel, its potential pitfalls and benefits, we propose five preliminary but key recommendations from which to move forward:

1. A global policy on medical travel should necessitate that quality information and communication are present during all stages of medical travel including decision-making, medical travel preparation, transportation, hospitalization, and follow-up care.
2. A global health access policy should ensure that medical travelers are able to receive appropriate social support during travel. This should be delineated explicitly in the policy, and should clarify financial details for a companion.
3. Respect for individual values will need to be aligned with existing international guidelines. This may require further consideration to ensure improved access does not require a tradeoff with cultural integrity.
4. Well-being is infrequently and inconsistently measured in the context of medical travel. Adequate approaches to well-being measurement should be developed in order to test the implications of a global policy on both short- and long-term health and well-being of medical travelers beyond immediate treatment outcomes.
5. More systematic and comparative evidence on the effects of medical travel, perhaps through the application of controlled trials, is required.

## Author Contributions

LZ, ÁM and KS were responsible for literature search and writing the original and submitted drafts of the manuscript. KR was the senior advisor and provided the initial framework plus subsequent recommendations.

## Conflict of Interest Statement

The authors declare that the research was conducted in the absence of any commercial or financial relationships that could be construed as a potential conflict of interest.
